# Corosolic acid increases the therapeutic effect of cisplatin on gastric cancer by regulating Gpx4-dependent ferroptosis

**DOI:** 10.20517/cdr.2025.94

**Published:** 2025-08-07

**Authors:** Liubing Lin, Jian Wang, Shun Sheng, Yanting Shen, Xiaolin Liu, Rongzhong Xu, Yong Li

**Affiliations:** ^1^Department of Gastroenterology, Shanghai Municipal Hospital of Traditional Chinese Medicine, Shanghai University of Traditional Chinese Medicine, Shanghai 200071, China.; ^2^Department of Traditional Chinese Medicine, Shenxin Community Health Service Center of Minhang District, Shanghai 201100, China.; ^3^Department of Oncology, Shanghai Municipal Hospital of Traditional Chinese Medicine, Shanghai University of Traditional Chinese Medicine, Shanghai 200071, China.; ^#^These authors contributed equally to this work.

**Keywords:** GC, cisplatin, corosolic acid, ferroptosis, Gpx4

## Abstract

**Aim:** Cisplatin serves as a primary chemotherapeutic agent in the treatment of gastric cancer (GC), but resistance to cisplatin-based chemotherapeutic regimens hampers its clinical application. Corosolic acid (CA), a natural triterpenoid, exhibits both anti-inflammatory and anti-cancer activities. However, the effect of CA on improving cisplatin resistance in GC remains unclear. The study primarily aimed to evaluate whether CA increases the therapeutic efficacy of cisplatin against GC and to reveal its underlying mechanism.

**Methods:** Cisplatin and CA were used to treat GC cells or cisplatin-resistant AGS cells (AGS-CR), and then cell viability, apoptosis, and growth were assessed using Cell Counting Kit-8, TdT-mediated dUTP nick end labeling, and clone formation assays, respectively. Glutathione peroxidase 4 (Gpx4) expression was measured through quantitative real-time PCR and western blotting assays.

**Results:** CA treatment induced a dose-dependent reduction in GC cell viability. The combination of cisplatin and CA resulted in enhanced cytotoxicity and pro-apoptotic effects compared to treatment with cisplatin alone. The effect of CA as a chemosensitizer in GC cells was damaged by a ferroptosis inhibitor, suggesting that CA decreased cisplatin chemoresistance by accelerating cancer cell ferroptosis. CA triggered cell ferroptosis by repressing Gpx4 expression in GC cells. Furthermore, elevated Gpx4 expression was significantly associated with poorer overall and disease-free survival.

**Conclusion:** CA has the potential to increase cisplatin chemosensitivity in GC, and Gpx4 may represent a promising therapeutic target for its treatment.

## INTRODUCTION

Gastric cancer (GC) ranks as the fifth most frequently diagnosed malignancy globally, with an estimated one million new cases in 2020. It is also the fourth leading cause of cancer-related mortality, with an estimated 770,000 deaths^[1,2]^. Owing to the deficiency of representative symptoms and effective markers, GC is typically diagnosed at an advanced stage, which results in a poor prognosis^[3]^. Over the past few decades, despite the great progress in molecular-targeted therapy, surgical resection and chemotherapy remain the mainstays of therapy for GC patients^[4,5]^. Cisplatin is an effective drug to treat GC, but resistance to cisplatin-based regimens hampers its clinical application. Numerous studies have investigated the molecular mechanisms underlying chemotherapy resistance by generating cisplatin-resistant GC cell lines, such as cisplatin-resistant AGS cells (AGS-CR). Studies on AGS-CR cells indicate a lower accumulation of cisplatin compared to sensitive cells, attributed to decreased drug uptake^[6]^. RNA-seq analysis of AGS-CR cells identified 189 differentially expressed genes (DEGs) primarily linked to cisplatin resistance-related molecular functions. DEG analysis further revealed enrichment across 23 metabolic pathways, with the inflammation-mediated chemokine and cytokine signaling pathway exhibiting the highest degree of enrichment^[7]^.

Radix Actinidiae chinensis (RAC), a traditional Chinese medicine, possesses a significant anti-cancer property against different types of tumors, such as GC^[8]^, colorectal cancer (CRC)^[9]^, and lung cancer^[10]^. RAC treatment inhibits inflammatory response and cell proliferation in renal cell carcinoma (RCC) mice and accelerates cancer cell apoptosis^[11]^. Ethanol extract of RAC accelerates CRC cell apoptosis by repressing Notch signaling^[12]^. Importantly, several bioactive ingredients have been identified in RAC. Oleanolic acid (OA), a bioactive triterpenoid in RAC, exhibits effective anti-tumor activity in various types of tumors^[13,14]^. OA derivatives (CDDO, CDDO-me, K73-03, *etc.*) improve their anti-tumor activity through increasing water solubility^[15-17]^. Corosolic acid (CA), another bioactive triterpenoid in RAC, could effectively inhibit cell proliferation and tumor growth in many types of tumors, such as GC, prostate cancer (PC), and hepatocellular carcinoma (HCC)^[18-20]^. Furthermore, CA could increase the responsiveness of cancer cells to 5fluorouracil (5FU) treatment^[21]^. However, the potential of CA to increase the therapeutic effect of cisplatin on GC remains poorly characterized.

Ferroptosis is a kind of non-apoptotic cell death marked by iron-dependent lipid peroxidation^[22]^. Ferroptosis plays a vital role in regulating tumor progression and chemoresistance^[23,24]^. Wang *et al.* showed that overactivation of the Wnt/β-catenin pathway inhibits ferroptosis through increasing Gpx4 expression, resulting in GC cell chemoresistance^[24]^. A recent study has demonstrated that CA sensitizes HCC cells to ferroptosis through decreasing glutathione (GSH) synthesis^[25]^. At present, several critical genes involved in ferroptosis have been identified, such as prostaglandin-endoperoxide synthase 2 (Ptgs2), acyl-CoA synthetase, long-chain family member 4 (Acsl4), Gpx4, NADPH oxidase 1 (Nox1), Ferritin heavy chain 1 (Fth1), and solute carrier family 7 member 11 (Slc7a11)^[26]^. Increased expression of Acsl4^[27]^, Ptgs2^[28]^, and Nox1^[29]^ contributes to inhibiting ferroptosis, while decreased expression of Gpx4^[30]^, Slc7a11^[31]^, and Fth1^[32]^ promotes ferroptosis. The study aimed to assess the chemosensitizing potential of CA in GC cells and reveal its underlying mechanisms.

## MATERIALS AND METHODS

### Gene expression profiling interactive analysis

Gpx4 expression and its association with overall survival (OS) and disease-free survival (DFS) were analyzed using the gene expression profiling interactive analysis (GEPIA) platform based on data from the cancer genome atlas-stomach adenocarcinoma (TCGA-STAD), which comprises 408 tumor and 211 normal gastric tissue samples.

### Cell culture

AGS and MKN-45 cells (The Shanghai Institute of Biochemistry and Cell Biology, Shanghai, China) were cultivated in DMEM/F12 (GIBCO, NY, USA) supplemented with 10% FBS (GIBCO) in a humidified 5% CO_2_ incubator. AGS-CR were established through persistent exposure of parental AGS cells to escalating concentrations of cisplatin for more than one year, as previously described^[7]^. AGS-CR cells were maintained in DMEM/F12 containing cisplatin to preserve their drug-resistant phenotype.

The pcDNA-Gpx4 recombinant plasmids, which encode full-length cDNA of Gpx4, were constructed to overexpress Gpx4 in GC cells. PolyFast Transfection Reagent (MedChem Express, NJ, USA) was applied to transfect the recombinant plasmids into cells when cells reached approximately 80% confluence.

To evaluate the cytotoxicity of cisplatin (MedChem Express) and CA (MedChem Express) to GC cells, AGS, MKN-45, or AGS-CR cells were treated with cisplatin (5, 15, 25, and 35 µm) and CA (0, 2, 5, 10, 20, 40, and 60 µm). Cell viability, clone formation ability, and cell apoptosis were evaluated through cell counting kit-8 (CCK-8), clone formation assay, TUNEL staining, and flow cytometry, respectively.

### CCK-8

Cell viability was assessed by the CCK-8 assay. Cells were seeded in 96-well plates (5 × 10^3^ cells per well) overnight, followed by treatment with cisplatin and CA for the specified duration. Subsequently, CCK-8 solution (10 μL, MedChem Express) was added to each well and incubated for 1 h, after which absorbance at 450 nm was measured using a VICTOR Nivo™ microplate reader (Univ-bio, Shanghai, China).

### Clone formation assay

AGS or AGS-CR cells (400 cells per well) were treated with cisplatin (35 µm) and/or CA (5 µm) for 3 days. Then, cells were cultured in DMEM/F12 medium for two weeks, allowing colony formation to reach visible levels. After washing four times with phosphate buffer saline (PBS), cells were treated with 4% paraformaldehyde (PFA) for fixation, followed by staining with 0.1% crystal violet. Colonies were observed with an inverted microscope (VEDENG, Shanghai, China).

### Apoptosis assay

Cell apoptosis was assessed using TUNEL and flow cytometry. In brief, AGS-CR cells were exposed to cisplatin (35 µm) and/or CA (5 µm) for 24 h, then fixed with 4% PFA and subsequently stained using TUNEL reagent (Solarbio, Beijing, China). TUNEL-positive cell quantification was performed using fluorescence microscopy (Leica). Apoptotic cell populations were additionally evaluated using the Annexin V-FITC/PI detection kit (MedChem Express) in conjunction with a flow cytometer (BD Biosciences Inc.)

### Fluorescein diacetate staining

AGS-CR cells were exposed to cisplatin (35 µm) and/or CA (5 µm) or Ferrostatin-1 (Fer-1, a potent ferroptosis inhibitor, 1 μm) for 24 h, then fixed with 4% PFA and subsequently incubated with fluorescein diacetate (FDA) solution (Merck, MA, USA) for 20 min. FDA-positive cell quantification was performed using fluorescence microscopy

### qRT-PCR

Total RNA was collected with Trizol (Solarbio), followed by first-strand cDNA synthesis with M-MLV and Oligo(dT) primers (TaKaRa, Tokyo, Japan). Quantitative reverse transcription polymerase chain reaction (qRT-PCR) was carried out with SYBR Green qPCR mix on the 7500 real-time qPCR system (Applied Biosystems). Relative mRNA level was quantified through the 2^-ΔΔCt^ method^[33]^. β-actin was applied as a reference gene. All primers were listed in Supplementary Table 1.

### Western blot

Total protein was collected from GC cells with RIPA buffer (Sigma-Aldrich, MO, USA) and quantitated with a BCA protein assay kit (Abcam, CA, USA). Approximately 20 µg of protein were separated on 12% SDS-PAGE at an unchanging voltage of 50 V (concentration gel) or 110 V (separation gel). Subsequently, protein was transferred to PVDF membranes (Merck), and the membranes were sealed with 5% skimmed milk solution, washed thrice with PBS, and incubated with primary antibodies for 1 h and HRP-conjugated secondary antibodies (1:5,000, ab6728 or ab6721, Abcam) for 1-h at room temperature. The primary antibodies used in the study were Gpx4 (1:4,000, ab125066, Abcam), Ptgs2 (1:2,000, #35-8200, Thermo Fisher Scientific, MA, USA), Acsl4 (1:10,000, #ab155282, Abcam), Fth1 (1:1,000, #ab75972, Abcam), Nox1 (1:2,000, #PA5-38031, Thermo Fisher Scientific), Slc7a11 (1:4,000, ab175186, Abcam), and β-actin (1:5,000, #ab8226, Abcam). The immunoblots were visualized with an ECL detection reagent (Solarbio) on the Tanon 5200 system (Tanon, Shanghai, China).

### Reactive oxidative species assay

AGS and MKN-45 cells were treated with CA (5 µm). Twenty-four hours later, cells were incubated with C11-BODIPY (1 μm, Thermo Fisher Scientific) for 30 min at 37 °C. Lipid reactive oxidative species (ROS) level was tested with a flow cytometer (BD Biosciences, CA, USA).

### Assessment of iron, malondialdehyde, and GSH levels

AGS and MKN-45 cells were exposed to CA (5 µm). Twenty-four hours later, ferrous iron concentration, malondialdehyde (MDA), and GSH content were evaluated using an iron assay kit (ab83366), a MDA assay kit (ab118970), and a GSH assay kit (ab65322), respectively.

### Mouse xenograft tumor model

The work was approved by the Animal Ethics Committee of Shanghai Municipal Hospital of Traditional Chinese Medicine (No. 2023112) and conducted in compliance with the ARRIVE guidelines. BALB/c nude mice (Charles River, Shanghai, China) were maintained in pathogen-free rooms at 22-26 °C and 40%-60% humidity under a 12-h light-dark cycle. Mice had unrestricted access to standard chow and water. The GC xenograft model in mice was created by subcutaneously injecting AGS cells (5 × 10^6^ cells in 100 μL PBS) into the right flank (*n* = 5 per group). Upon the tumors reaching roughly 100 mm^3^ in volume, the mice were administered according to the specified schedule and dosage: 1) intraperitoneal (i.p.) injection of vehicle, 2) i.p. injection of cisplatin once a day for 2 weeks (5 mg/kg), 3) i.p. injection of cisplatin once a day for 2 weeks (5 mg/kg) and CA once every 2 days (20 mg/kg). Tumor volume and body weight were surveyed at the specified intervals. Throughout the experiment, mice exhibiting severe illness or approaching death were humanely euthanized via cervical dislocation under deep anesthesia with pentobarbital sodium (100 mg/kg).

### Statistics

Results from three biologically independent experiments were presented as mean ± standard deviation. Statistical comparisons were conducted through Student’s *t* test or one-way analysis of variance (ANOVA) tests via GraphPad Prism 7.0 (GraphPad Software, CA, USA), with *P* < 0.05 denoting significance.

## RESULTS

### CA sensitized GC cells to cisplatin treatment

**Figure 1:**
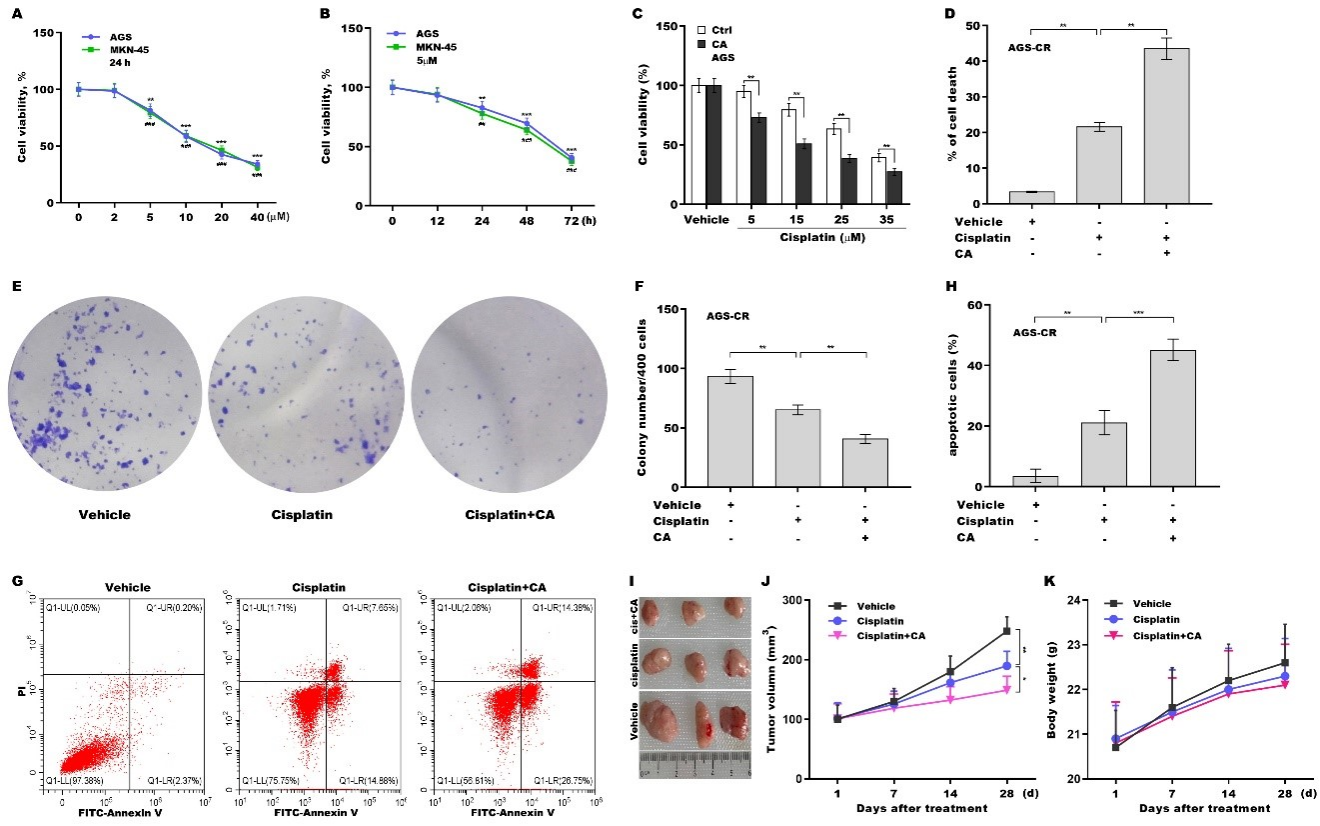
CA sensitized GC cells to cisplatin treatment. (A) AGS and MKN-45 cells were exposed to different concentrations of CA (0, 2, 5, 10, 20, and 40 μm) for 24 h, and cell viability was measured using CCK-8 (*n* = 3). Statistical significance was assessed using one-way ANOVA followed by Dunnett’s post-hoc test (AGS: **P < 0.01, ***P < 0.001. MKN-45: ##*P* < 0.01, ###*P* < 0.001); (B) AGS and MKN-45 cells were exposed to CA (5 µm) for different times (0, 12, 24, 48, and 72 h), and cell viability was assessed using CCK-8 (*n* = 3). Statistical significance was assessed using one-way ANOVA followed by Dunnett’s post-hoc test (AGS: **P < 0.01, ***P < 0.001. MKN-45: ##*P* < 0.01, ###*P* < 0.001); (C) AGS cells were exposed to CA (20 µm) and different concentrations of cisplatin (0, 5, 15, 25, and 35 µm) for 24 h, and cell viability was assessed using the CCK-8 assay (*n* = 3). Statistical significance was assessed using multiple *t*-tests; (D) AGS-CR cells were exposed to 5 µm of CA and 35 µm of cisplatin for 24 h, after which cell death was assessed using the CCK-8 assay (*n* = 3). Statistical significance was assessed using one-way ANOVA followed by Dunnett’s post-hoc test. AGS-CR cells were treated with 5 µm of CA and 35 µm of cisplatin for 3 days, and then clone formation assay (E) and quantitative analysis (F) were carried out (*n* = 3). Statistical significance was assessed using one-way ANOVA followed by Dunnett’s post-hoc test. AGS-CR cells were treated with 5 µm of CA and 35 µm of cisplatin for 24 h, after which flow cytometry (G) and quantitative analysis (H) were carried out (*n* = 3). Statistical significance was assessed using one-way ANOVA followed by Dunnett’s post-hoc test; (I-K) To assess the effect of CA on cisplatin resistance and its safety *in vivo*, a mouse xenograft model was developed by subcutaneously injecting AGS cells into nude mice. Once tumor volume approached 100 mm^3^, the mice with tumors were administered either vehicle, cisplatin, or combination treatment with cisplatin and CA, and tumor growth was monitored (I and J) and body weight was measured (K) (*n* = 3). Statistical significance was assessed using one-way ANOVA followed by Dunnett’s post-hoc test. *p < 0.05, **P < 0.01, ***P < 0.001. CA: Corosolic acid; GC: gastric cancer; CCK-8: cell counting kit-8; ANOVA: analysis of variance; CR: cisplatin-resistant.

To explore the effect of CA on increasing cisplatin sensitivity in GC cells, the cytotoxicity of CA to GC cells (AGS and MKN-45 cells) was first evaluated. Figure 1A reveals that AGS and MKN-45 cell viability were decreased by approximately 18% and 20%, respectively, at a concentration of 5 µm of CA and further decreased in a concentration-dependent manner. 5 µm of CA were not toxic to a normal gastric epithelial cell line [Supplementary Figure 1A] and thus were used in subsequent experiments. CA also decreased GC cell viability in a time-dependent manner in AGS and MKN-45 cells [Figure 1B]. Importantly, combination treatment with cisplatin and CA reduced AGS [Figure 1C] and MKN-45 [Supplementary Figure 1B] cell viability more effectively than cisplatin alone. Then the effect of CA on AGS-CR cell viability, proliferation, and apoptosis was assessed using CCK-8, clone formation, TUNEL, and flow cytometry assays, respectively. Combination treatment with cisplatin and CA decreased AGS-CR cell viability [Figure 1D] and clone formation capacity [Figure 1E and F] more effectively than cisplatin alone. The results from TUNEL [Supplementary Figure 1C and D] and flow cytometry analyses [Figure 1G and H] showed that the combination treatment accelerated AGS-CR cell apoptosis more effectively than cisplatin alone. Furthermore, a mouse subcutaneous xenograft model was generated to assess the effect of CA on cisplatin resistance and to evaluate its safety *in vivo*. The combination of cisplatin and CA more effectively slowed down tumor growth compared to cisplatin alone [Figure 1I and J]. There was no notable difference in body weight across the groups [Figure 1K]. These results demonstrate that CA increases cisplatin sensitivity in GC cells, cisplatin-resistant GC cells, and *in vivo* xenograft models.

### Ferroptosis inhibition decreased the effect of CA as a chemosensitizer in GC cells

**Figure 2:**
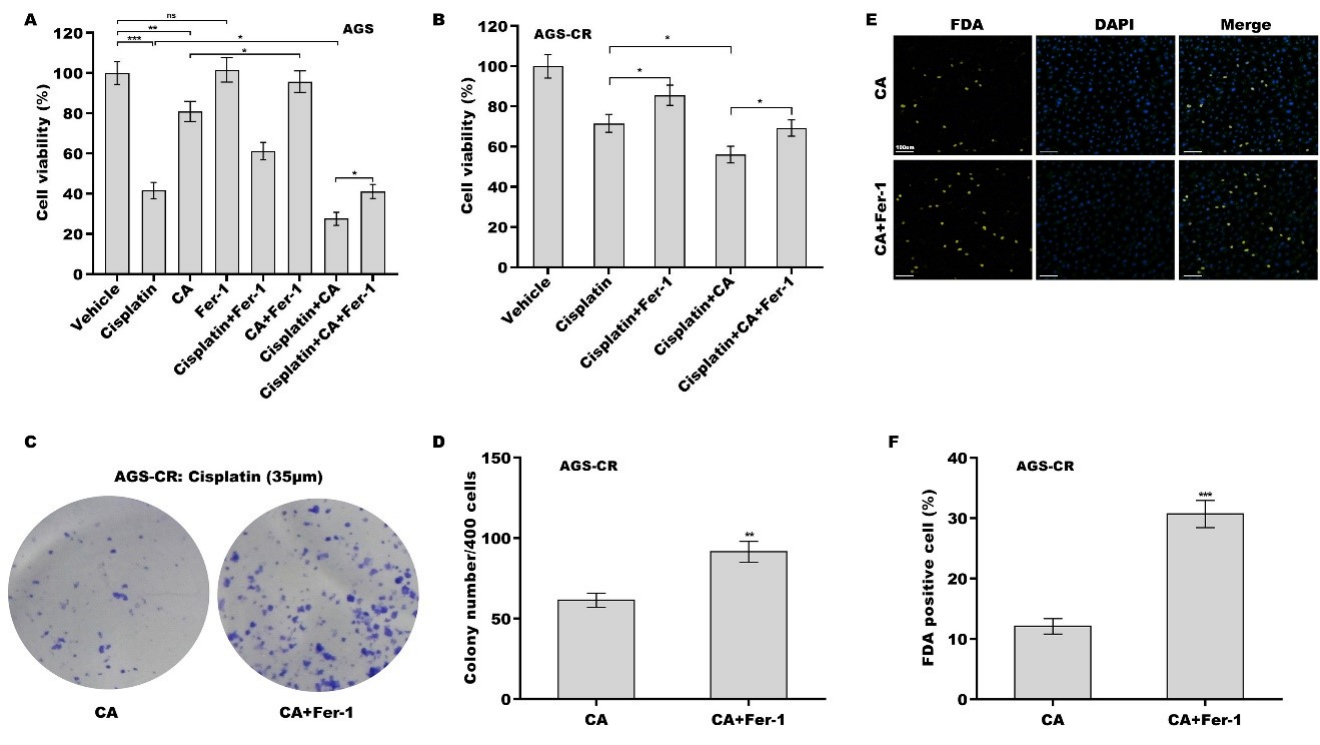
The effect of CA as a chemosensitizer in GC cells was damaged by Fer-1. AGS (A) and AGS-CR (B) cells were exposed to cisplatin (35 µm) and CA (5 µm) or Ferrostatin-1 (Fer-1, 1 μm) for 24 h, and then cell viability was measured using the CCK-8 assay (*n* = 3). Statistical significance was assessed using one-way ANOVA followed by Tukey’s post-hoc test. AGS-CR cells were exposed to cisplatin (35 µm) and CA (5 µm) in the presence or absence of Fer-1 (1 μm), and then clone formation assay (C) and quantitative analysis (D) were carried out (*n* = 3). Statistical significance was assessed using Student’s *t* test. AGS-CR cells were exposed to cisplatin (35 µm) and CA (5 µm) in the presence or absence of Fer-1 (1 μm), and then FDA staining (E) and quantitative analysis (F) were performed (*n* = 3). Statistical significance was assessed using Student’s *t* test. *P < 0.05, **P < 0.01, ***P < 0.001. CA: Corosolic acid; GC: gastric cancer; CR: cisplatin-resistant; CCK-8: cell counting kit-8; FDA: fluorescein diacetate.

Given the vital role of ferroptosis in cisplatin resistance^[24,34,35]^, the effect of ferroptosis on cisplatin- or CA-induced GC cell death was next assessed. For this purpose, a ferroptosis-specific inhibitor, Fer-1, was used to treat GC cells in the presence of CA, cisplatin, or both. Although Fer-1 alone did not affect cell viability, Fer-1 alleviated the cytotoxicity of cisplatin and CA to AGS cells [Figure 2A] and MKN-45 cels [Supplementary Figure 1E]. Fer-1 further alleviated the cytotoxicity of cisplatin to AGS-CR cells [Figure 2B], indicating that cisplatin exhibited cytotoxicity to GC cells by triggering ferroptosis. Combination treatment with cisplatin and CA decreased AGS [Figure 2A] and AGS-CR [Figure 2B] cell viability more effectively than cisplatin alone, whereas these effects were reversed by Fer-1 in these cells. The results from the clone formation assay and FDA staining showed that Fer-1 treatment significantly increased the clone formation capacity [Figure 2C and D] and cell viability [Figure 2E and F] in AGS-CR cells. These data suggest that CA might function as a chemosensitizer in GC cells, at least in part by accelerating ferroptosis.

### CA triggered GC cell ferroptosis by inhibiting Gpx4 expression

**Figure 3:**
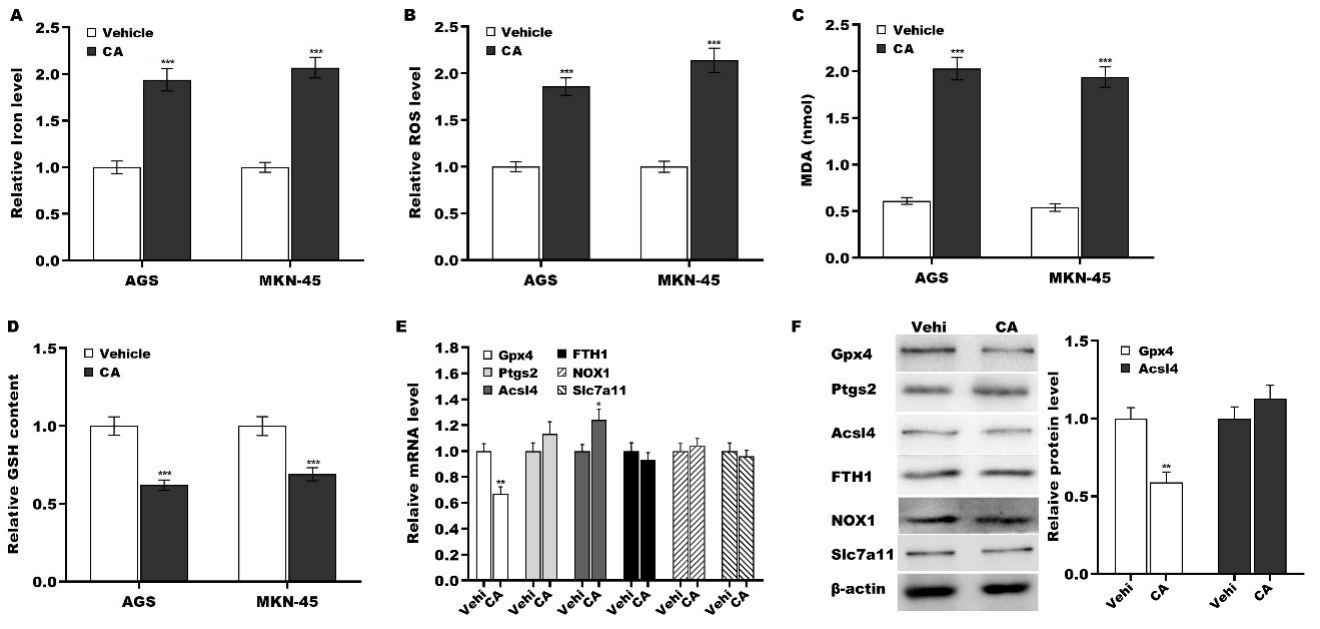
CA triggered GC cell ferroptosis by inhibiting Gpx4 expression. AGS and MKN-45 cells were exposed to 5 µm of CA for 24 h, and the levels of iron (A), ROS (B), MDA (C), and GSH (D) were assessed using a commercial assay kit. AGS cells were treated with 5 µm of CA for 24 h, and the mRNA (E) and protein (F) levels of Gpx4, Ptgs2, Acsl4, Fth1, Nox1, and Slc7a11 were assessed using qRT-PCR and western blot, respectively. All statistical analyses were assessed using Student’s *t* test (*n* = 3). **P < 0.01, ***P < 0.001. CA: Corosolic acid; GC: gastric cancer; Gpx4: glutathione peroxidase 4; Ptgs2: prostaglandin-endoperoxide synthase 2; Fth1: ferritin heavy chain 1; Nox1: NADPH oxidase 1; Slc7a11: solute carrier family 7 member 11; qRT-PCR: quantitative reverse transcription polymerase chain reaction.

To define the biological effect of CA on GC cell ferroptosis, CA was applied to treat GC cells, and then ferroptosis was evaluated by measuring iron, ROS, MDA, and GSH levels, as previously described^[36]^. Figure 3A-D reveals that CA treatment resulted in significantly increased levels of iron, ROS, and MDA and decreased levels of GSH in AGS and MKN-45 cells, indicating that CA promoted GC cell ferroptosis. To uncover the molecular mechanism underlying GC cell ferroptosis triggered by CA, the expression of key molecules involved in ferroptosis (Gpx4, Ptgs2, Acsl4, Fth1, Nox1, and Slc7a11)^[26,27,30,37]^ was measured in GC cells following treatment with vehicle or CA. Compared to the vehicle control, CA treatment significantly downregulated Gpx4 mRNA expression while simultaneously upregulating Ptgs2 transcripts [Figure 3E]. At the protein level, CA markedly suppressed Gpx4 expression, while Ptgs2 levels remained unchanged [Figure 3F].

### High Gpx4 expression predicted poor OS and DFS

**Figure 4:**
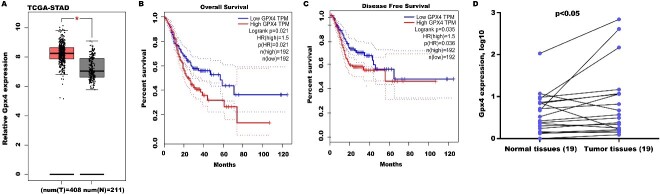
High Gpx4 expression predicted a poor OS and DFS. (A) Gpx4 expression was analyzed in the TCGA-STAD database through the GEPIA tool. STAD tissues (*n* = 408) were displayed as red and normal tissues (*n* = 211) were displayed as grey. The GEPIA tool was applied to analyze the correlation of Gpx4 expression with OS (B) and DFS (C) in the TCGA-STAD database; (D) qRT-PCR analysis of Gpx4 mRNA level in 19 pairs of GC tissues and normal tissues. Statistical significance was assessed using Student’s *t* test (*n* = 19). *P < 0.05, **P < 0.01. Gpx4: Glutathione peroxidase 4; OS: overall survival; DFS: disease-free survival; TCGA-STAD: the cancer genome atlas-stomach adenocarcinoma; GEPIA: gene expression profiling interactive analysis; qRT-PCR: quantitative reverse transcription polymerase chain reaction.

To define the function of Gpx4 on GC progression, Gpx4 expression was analyzed in the TCGA-STAD database. Figure 4A shows that Gpx4 levels were prominently upregulated in GC tissues compared with normal tissues. Moreover, high Gpx4 expression predicted poor 10-year OS [Figure 4B] and DFS [Figure 4C]. Gpx4 expression was also assayed in 19 GC tissues and matched normal tissues. Figure 4D reveals that Gpx4 expression was also upregulated in most GC tissues compared with matched normal tissues.

### CA increased cisplatin sensitivity by regulating Gpx4

**Figure 5:**
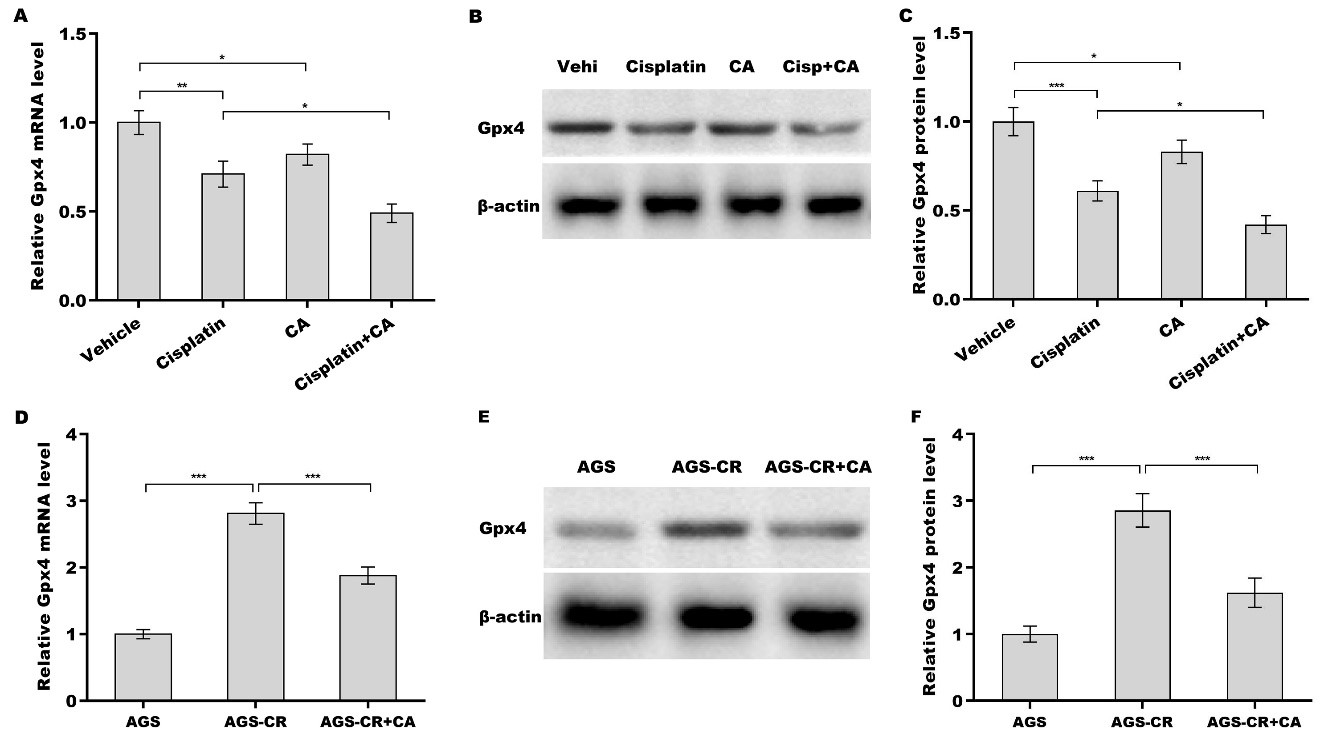
CA decreased Gpx4 expression. After treatment with cisplatin (35 µm) alone, CA (5 µm) alone, or combination treatment with cisplatin (35 µm) and CA (5 µm) in AGS cells, Gpx4 mRNA (A) and protein (B and C) levels were assessed using qRT-PCR and western blot, respectively. qRT-PCR (D) and western blot (E and F) analyses of Gpx4 expression in AGS, AGS-CR, and CA-treated AGS-CR cells. All statistical analyses were assessed using one-way ANOVA followed by Tukey’s post-hoc test (*n* = 3). *P < 0.05, **P < 0.01, ***P < 0.001. CA: Corosolic acid; Gpx4: glutathione peroxidase 4; qRT-PCR: quantitative reverse transcription polymerase chain reaction; CR: cisplatin-resistant; ANOVA: analysis of variance.

**Figure 6:**
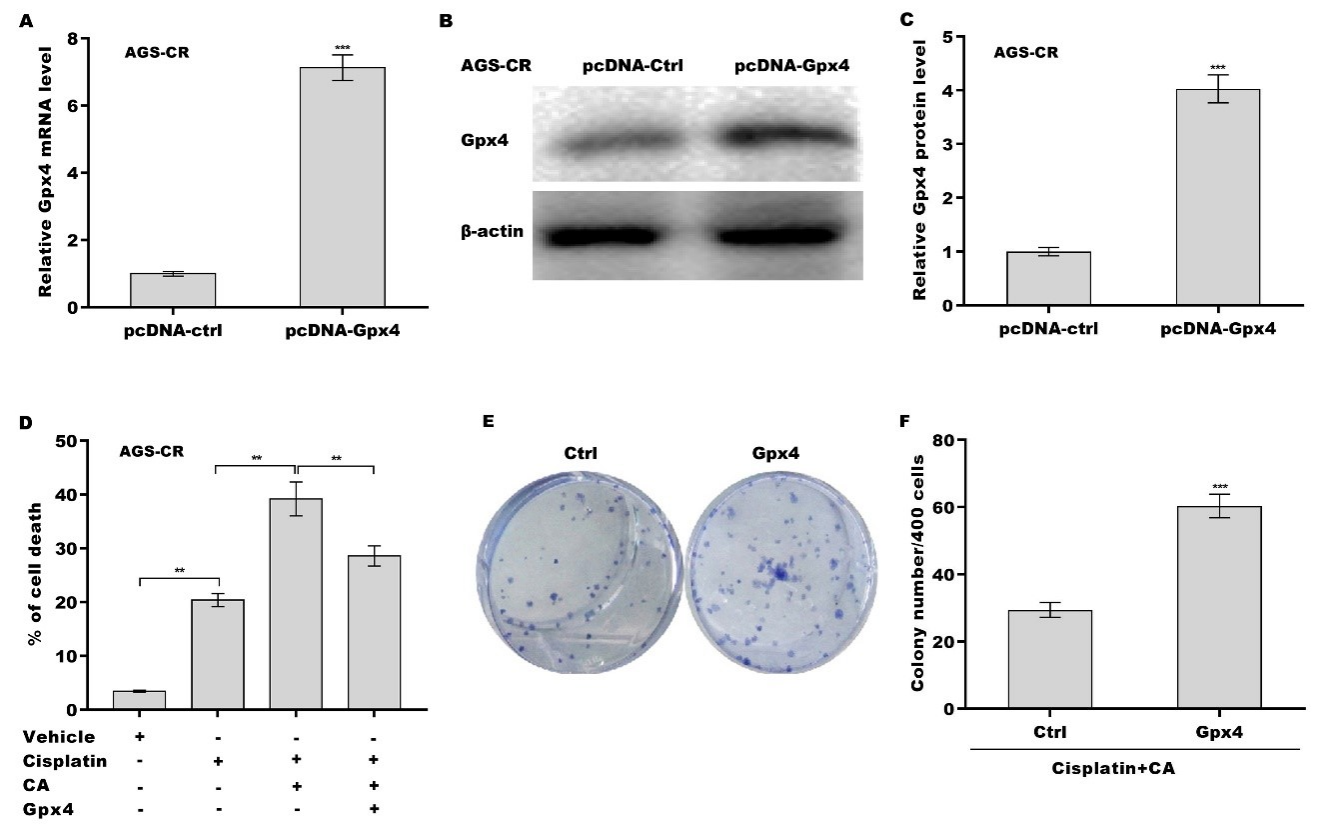
CA increased cisplatin sensitivity by regulating Gpx4. AGS-CR cells were transfected with recombinant plasmids of pcDNA-Gpx4 (1.5 µg) for 48 h, and the Gpx4 mRNA (A) and protein (B and C) levels were assessed using qRT-PCR and western blot, respectively; (D) AGS-CR cells were treated with cisplatin (35 µm), CA (5 µm), and pcDNA-Gpx4 and cell death was assessed using CCK-8; (E and F) AGS-CR cells were treated with cisplatin (35 µm) and CA (5 µm) in the presence or absence of Gpx4 overexpression and clone formation ability was assessed. All statistical analyses, except for panel D (analyzed using one-way ANOVA followed by Tukey’s post-hoc test), were performed using Student’s *t*-test (*n* = 3). **P < 0.01, ***P < 0.001. CA: Corosolic acid; Gpx4: glutathione peroxidase 4; qRT-PCR: quantitative reverse transcription polymerase chain reaction; CR: cisplatin-resistant; CCK-8: cell counting kit-8; ANOVA: analysis of variance.

Finally, we investigated whether CA inhibited Gpx4 expression and thus increased cisplatin sensitivity in GC cells. Figure 5A-C shows that treatment with either cisplatin or CA alone obviously decreased Gpx4 expression at the mRNA and protein levels, and the inhibitory effect was reinforced through combination treatment with cisplatin and CA. Gpx4 expression level in AGS-CR cells was enhanced compared with AGS cells, whereas CA repressed Gpx4 expression in AGS-CR cells [Figure 5D-F], indicating that CA sensitized GC cells to cisplatin, possibly by inhibiting Gpx4 expression. To clarify Gpx4’s involvement in CA-mediated enhancement of cisplatin sensitivity, Gpx4 was overexpressed in AGS-CR cells, followed by evaluation of cell viability before and after its overexpression. As expected, Gpx4 overexpression in AGS-CR cells [Figure 6A-C] decreased cell death [Figure 6D] and increased clone formation ability [Figure 6E and F], thereby counteracting the effects of CA on sensitizing AGS-CR cells to cisplatin.

## DISCUSSION

GC is typically diagnosed at an advanced stage, which leads to a poor prognosis^[3,38]^. Cisplatin-based chemotherapy is one of the most broadly applied regimens to treat advanced patients with GC^[39,40]^. Unfortunately, almost all patients ultimately develop acquired resistance during the course of chemotherapy^[41]^. The mechanisms underlying cisplatin resistance are gradually being elucidated. For example, Cisplatin induces cytotoxicity by forming Platinum (Pt)-DNA adducts, which interfere with DNA replication and transcription. However, cancer cells may develop resistance by enhancing DNA repair mechanisms, especially nucleotide excision repair^[42,43]^. Cisplatin enters cells through copper transporters(CTR1), but resistant cells may downregulate CTR1, decreasing drug uptake^[44]^. An increasing number of studies highlight the potential of combination therapy in cancer treatment. In the study, we demonstrated that, (1) CA sensitized GC cells to cisplatin treatment; (2) Ferroptosis inhibition damaged the effect of CA as a chemosensitizer; (3) CA triggered GC cell ferroptosis by repressing Gpx4 expression; (4) High Gpx4 expression predicted a poor OS and DFS in patients with GC; and (5) CA increased cisplatin sensitivity by regulating Gpx4. These results reveal that CA alleviates cisplatin resistance in GC by regulating Gpx4-dependent ferroptosis.

Plants have been a credible source of medication for thousands of years because of their efficiency and low toxicity in the treatment of diseases^[45,46]^. Several bioactive monomers in medicinal plants have been identified as effective antineoplastic drugs, such as paclitaxel, vincristine, homoharringtonine, and cytarabine^[47,48]^. RAC is a traditional Chinese medicine, possessing a significant anti-tumor property. Ethanol extract of RAC inhibits cancer cell proliferation and invasion in many types of tumors^[11,12]^. CA is a bioactive triterpenoid in RAC and exhibits an effective anti-tumor property in GC, PC, and HCC^[18-20]^. Cheng *et al.* demonstrated that CA accelerates GC cell apoptosis by inhibiting nuclear factor kappa B (NF-κB) p65 expression and NF-κB signaling activation^[18]^. Moreover, CA could increase the chemosensitivity of GC cells to 5-FU by inhibiting the mammalian target of rapamycin (mTOR) and activating adenosine monophosphateactivated protein kinase (AMPK) signaling^[21,49]^. A recent study revealed that CA promotes HCC cell ferroptosis, a ROS-dependent cell death form, by inhibiting GSH synthesis^[25]^. CA also promotes oxidative stress in non-small cell lung cancer^[50]^. Nevertheless, most natural ingredients including CA^[51-54]^ in medicinal plants possess antioxidative activity^[55-57]^. Perhaps the effects of natural ingredients on regulating oxidative stress and cell viability may vary depending on drug concentration or cell type. It is very meaningful to investigate the role of CA in regulating cisplatin sensitivity and oxidative stress in GC cells.

Ferroptosis is an iron-dependent cell death mechanism characterized by ROS overproduction^[22]^. Upregulated Gpx4 inhibits ferroptosis in cancer cells to acquire cisplatin resistance^[58]^. Inhibition of nuclear factor erythroid 2-related factor 2 (Nrf2) upregulates ROS levels, potentiating ferroptosis and enhancing cisplatin sensitivity^[59]^. Therefore, initiating ferroptosis is emerging as a potential strategy for cancer therapy^[60,61]^. An increasing number of ferroptosis inducers are being identified and developed. As classical ferroptosis inducers, erastin and RLS3, possess potent anti-cancer capacity in different types of tumors, such as HCC^[62]^, melanoma^[63]^, GC^[64]^, and breast cancer^[65]^. Moreover, ferroptosis inducers can also overcome resistance to several first-line chemotherapy drugs. For example, RSL3 increases the anti-cancer effectiveness of cisplatin by regulating Gpx4-dependent ferroptosis^[66]^. Erastin reverses the resistance of head and neck cancer cells to cisplatin by inducing ROS overproduction^[67]^. Previous studies have demonstrated that CD44 inhibits ferroptosis by stabilizing Slc7a11, maintaining cystine uptake and GSH levels, and suppressing lipid ROS accumulation^[68-70]^. CD44-positive gastric cancer stem cells (CSCs) are less susceptible to ferroptosis, contributing to their survival and resistance to treatment^[71]^. Therefore, it is of interest to investigate whether CA regulates CD44 expression to inhibit ferroptosis resistance-mediated chemoresistance.

It is worth noting that ROS plays important roles in cancer cell survival because ROS overproduction is a key factor responsible for apoptosis^[72]^, ferroptosis^[73]^, and pyroptosis^[74]^. Paradoxically, low ROS levels are essential to maintaining cell and tissue homeostasis and cancer stem cell survival^[75,76]^, which is a major cause of tumor chemotherapy resistance. Therefore, CA increases the therapeutic effect of cisplatin on GC, at least in part, by decreasing Gpx4 expression and resulting in ROS overproduction and ferroptosis.

This study has three major limitations that can be addressed in future research: (1) Given the roles of ROS in multiple cell death modalities (apoptosis, ferroptosis, pyroptosis, autophagy, *etc.*), it is necessary to investigate whether CA regulates other cell death mechanisms besides ferroptosis; (2) Although current findings have demonstrated that CA treatment significantly downregulates Gpx4 expression at both the mRNA and protein levels, elucidating the molecular mechanism underlying CA-mediated suppression of Gpx4 expression remains of considerable importance; (3) In many experiments, cell viability and apoptosis were assessed 24 h after drug exposure. Expanding the analysis to additional time points (e.g., 48 or 72 h) could further enhance the reliability and persuasiveness of the findings.

### Conclusion

CA increases cisplatin sensitivity in GC by regulating Gpx4-dependent ferroptosis, indicating its therapeutic potential in augmenting cisplatin efficacy and establishing Gpx4 as a promising molecular target for GC treatment.

## DECLARATIONS

### Availability of data and materials

The GEPIA tool (http://gepia.cancer-pku.cn/) was applied to analyze the correlation of Gpx4 expression with OS and DFS in the TCGA-STAD database. The data supporting the results of this study are available from the corresponding author (Yong Li) upon reasonable request.

### Copyright

© The Author(s) 2025.
