# Supplementary material for: Corosolic acid increases the therapeutic effect of cisplatin on gastric cancer by regulating Gpx4-dependent ferroptosis
Source: Cancer Drug Resist. 2025 Aug 7;8:40. doi: 10.20517/cdr.2025.94 (PMC12366429; doi:10.20517/cdr.2025.94)
Supplement: Supplementary file 1 [file cdr-8-40-SupplementaryMaterials.pdf]

Corosolic acid increases the therapeutic effect of cisplatin on gastric cancer by regulating Gpx4-dependent ferroptosis

Liubing Lin[1,#], Jian Wang[1,#], Shun Sheng[1], Yanting Shen[1,2], Xiaolin Liu[1], Rongzhong Xu[3], Yong Li[1]

[1]Department of Gastroenterology, Shanghai Municipal Hospital of Traditional Chinese Medicine, Shanghai University of Traditional Chinese Medicine, Shanghai 200071, China.

[2]Department of Traditional Chinese Medicine, Shenxin Community Health Service Center of Minhang District, Shanghai 201100, China.

[3]Department of Oncology, Shanghai Municipal Hospital of Traditional Chinese Medicine, Shanghai University of Traditional Chinese Medicine, Shanghai, 200071, China.

[#]These authors contributed equally to this work.

Correspondence to: Dr. Rongzhong Xu, Shanghai Municipal Hospital of Traditional Chinese Medicine, Shanghai University of Traditional Chinese Medicine, 274 Middle Zhijiang Road, Jing'an District, Shanghai, China 200071. E-mail: 18918036209@163.com; Dr. Yong Li, Shanghai Municipal Hospital of Traditional Chinese Medicine, Shanghai University of Traditional Chinese Medicine, 274 Middle Zhijiang Road, Jing'an District, Shanghai, China 200071. E-mail: liyong@shutcm.edu.cn

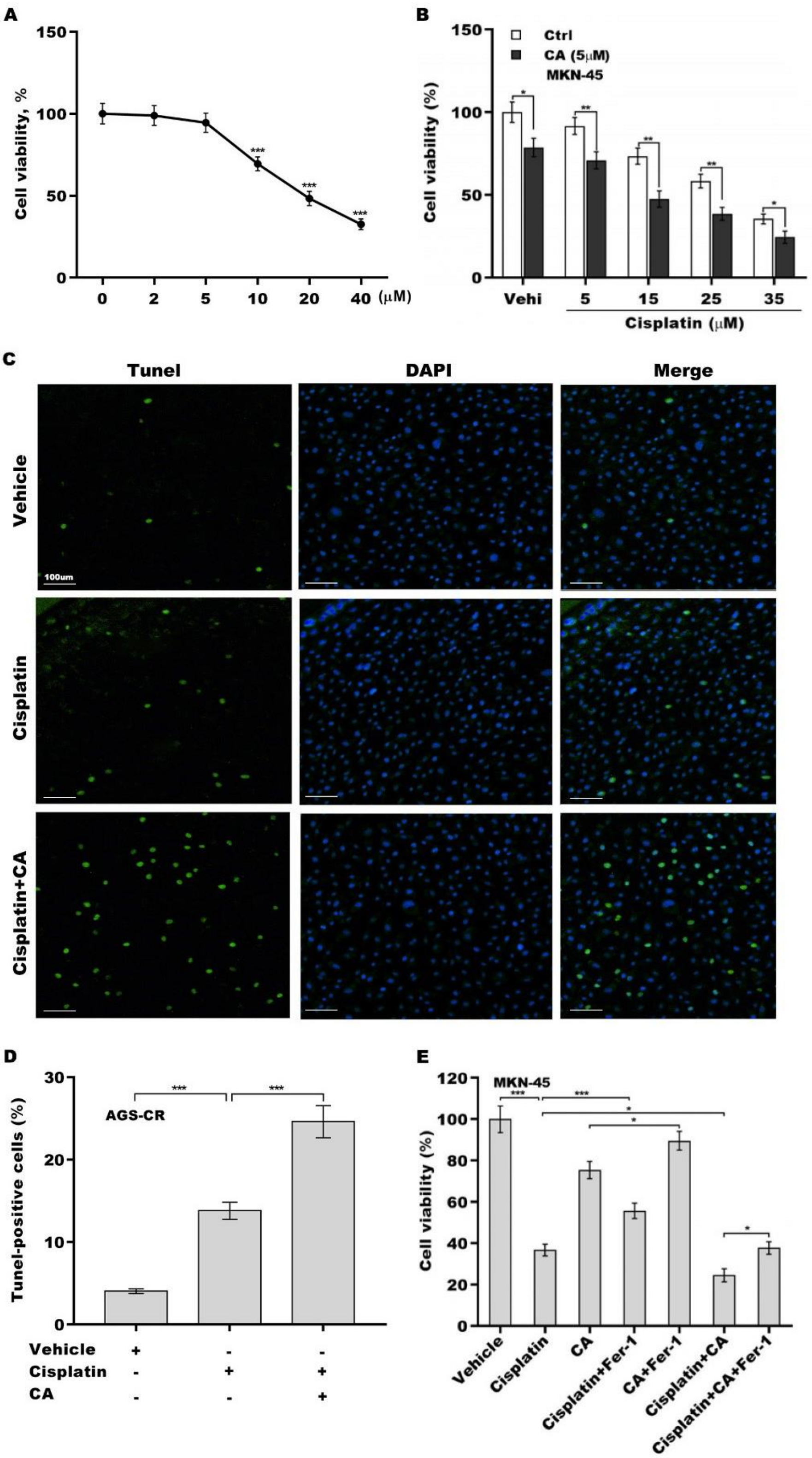

Supplementary Figure 1. (A) Normal gastric epithelial cells were treated with different concentrations of CA (0, 2, 5, 10, 20, and 40 μm) for 24 h and cell viability was measured using CCK-8 (*n* = 3). Statistical significance was assessed using one-way ANOVA followed by Dunnett's post-hoc test. (B) MKN-45 cells were

treated with CA (5 μm) and cisplatin (0, 5, 15, 25, and 35 μm) for 24 h, and cell viability was assessed using the CCK-8 assay (*n* = 3). Statistical significance was assessed using multiple *t*-tests. (C) AGS-CR cells were treated with cisplatin (35 μm) alone or in combination with CA (5μm) for 24 h, and cell viability was assessed using the CCK-8 assay. AGS-CR cells were treated with 5 μm of CA and 35 μm of cisplatin for 24 h, after which TUNEL (D) and quantitative analysis (E) were carried out. Statistical significance was assessed using one-way ANOVA followed by Tukey's post-hoc test (*n* = 3). *$P < 0.05$, **$P < 0.01$, ***$P < 0.001$.

Supplementary Table 1. Primer sequence used in the study

Gene	Sense, 5'-3'	Anti-sense, 5'-3'
Gpx4	GAACTTCACCAAGTTCCTCATCG	TGGGGCAGGTCCTTCTCTATC
Ptgs2	GACTCCCTTGGGTGTCAAAGG	AAAACTGATGCGTGAAGTGCTG
Acsl4	GGATTGGATATTCTTCTCCGCTT	ATTCATCTCTTGGACTTTGCTCAT
Fth1	ACTGACAAAAATGACCCCCAT	CAAAGAGATATTCCGCCAAGC
Nox1	GTCACCCCCTTTGCTTCTATCT	TTTGCCTAATTCCTCCATCTCC
Slc7a11	CCATGAACGGTGGTGTGTTTG	TAGAGGAGTGTGCTTGCGGAC
β-actin	CCGTTGCCCTGAGGCTCTTTT	TGCGGATGTCCACGTCACACT
